# Touch Accelerates Visual Awareness

**DOI:** 10.1177/2041669516686986

**Published:** 2017-01-01

**Authors:** Claudia Lunghi, Luca Lo Verde, David Alais

**Affiliations:** Department of Translational Research and New Technologies in Medicine and Surgery, University of Pisa, Italy; Institute of Neuroscience, CNR, Pisa, Italy; Institute of Neuroscience, CNR, Pisa, Italy; Department NEUROFARBA, University of Florence, Italy; School of Psychology, University of Sydney, NSW, Australia

**Keywords:** multisensory/cross-modal processing, psychophysics, rivalry/bistability, visuo-haptic interactions

## Abstract

To efficiently interact with the external environment, our nervous system combines information arising from different sensory modalities. Recent evidence suggests that cross-modal interactions can be automatic and even unconscious, reflecting the ecological relevance of cross-modal processing. Here, we use continuous flash suppression (CFS) to directly investigate whether haptic signals can interact with visual signals outside of visual awareness. We measured suppression durations of visual gratings rendered invisible by CFS either during visual stimulation alone or during visuo-haptic stimulation. We found that active exploration of a haptic grating congruent in orientation with the suppressed visual grating reduced suppression durations both compared with visual-only stimulation and to incongruent visuo-haptic stimulation. We also found that the facilitatory effect of touch on visual suppression disappeared when the visual and haptic gratings were mismatched in either spatial frequency or orientation. Together, these results demonstrate that congruent touch can accelerate the rise to consciousness of a suppressed visual stimulus and that this unconscious cross-modal interaction depends on visuo-haptic congruency. Furthermore, since CFS suppression is thought to occur early in visual cortical processing, our data reinforce the evidence suggesting that visuo-haptic interactions can occur at the earliest stages of cortical processing.

## Introduction

Combining information arising from different sensory modalities is essential to interact efficiently with the environment (Alais, Newell, & Mamassian, 2010). While multisensory integration—the merging the information from different modalities in a single percept —is not mandatory (Ernst & Banks, 2002; Hillis, Ernst, Banks, & Landy, 2002) and relies on attention (van Ee, van Boxtel, Parker, & Alais, 2009) and awareness (for a review on multimodal awareness, see Deroy, Chen, & Spence, 2014), cross-modal interactions might be more automatic. Cross-modal interactions occur when a signal from one sensory modality is modulated by a signal from another modality. Growing evidence suggests that cross-modal interactions between vision and other modalities can occur outside of visual awareness, such as when a visual stimulus presented to one eye undergoes a temporary suppression due to a conflicting image presented to the other eye (reviewed in Deroy et al., 2016; Faivre, Salomon, & Blanke, 2015). Studying such “unconscious” cross-modal interactions is important for understanding both cross-modal processing and human awareness. Regarding cross-modal processing, unconscious unisensory processing (e.g., visual) is limited mainly to the earliest stages of sensory processing (e.g., the primary visual cortex, Sterzer, Stein, Ludwig, Rothkirch, & Hesselmann, 2014). Thus, any demonstration of cross-modal interactions occurring outside of unisensory awareness would imply that primary sensory cortices should not be considered as uniquely unisensory but rather as areas that receive inputs from other sensory modalities. As for models of human awareness, cross-modal interactions outside of unisensory awareness have important theoretical implications because it would challenge the proposal that cross-modal binding requires conscious processing of the unisensory stimuli (Baars, 2002).

Two psychophysical paradigms are particularly efficient in inducing interocular suppression: binocular rivalry, and its variant continuous flash suppression (CFS). Binocular rivalry occurs when unrelated images are presented at the same time onto homologous portions of the two retinae. The conflicting dichoptic stimulation prevents binocular integration and triggers competing interocular inhibition processes, resulting in alternating suppression of each eye and thus fluctuations in visual perception between the two monocular images (Alais & Blake, 2005; Blake & Logothetis, 2002). CFS occurs when one eye is presented with a static visual stimulus, while the other one is presented with a dynamic mask in which random high-contrast patterns (known as “mondrians”) are flashed sequentially, typically at a frequency of 10 Hz (Tsuchiya and Koch, 2005). Dynamic patterns are known to predominate during binocular rivalry and CFS exploits this characteristic using flickering Mondrians to produce strong suppressive power and consequently long-lasting periods of suppression compared with binocular rivalry (Tsuchiya, Koch, Gilroy, & Blake, 2006). An advantage of CFS is that while suppression epochs in binocular rivalry are highly variable in duration and the dominant percept at onset is unpredictable, CFS allows a more predictable suppression: if no transients occur in the eye presented with the static image, the dynamic mask will immediately suppress the other stimulus for several seconds.

Evidence from several recent studies has shown that during either binocular rivalry or CFS, non-visual sensory modalities (e.g., auditory; Conrad, Bartels, Kleiner, & Noppeney, 2010; Lunghi, Morrone, & Alais, 2014, haptic; Lunghi & Alais, 2013, 2015; Lunghi, Binda, & Morrone, 2010; Lunghi & Morrone, 2013; Lunghi et al., 2014, olfactory; Zhou, Jiang, He, & Chen, 2010, proprioceptive; Salomon, Lim, Herbelin, Hesselmann, & Blanke, 2013, vestibular; Salomon, Kaliuzhna, Herbelin, & Blanke, 2015, and bodily; Saletu, Anderer, Kinsperger, Grünberger, & Sieghart, 1988, signals) are able to interact with the suppressed visual stimulus (reviewed in Deroy et al., 2016). In particular, it has been shown that during binocular rivalry, unambiguous haptic stimulation promotes dominance of the congruent visual stimulus independently of awareness: touch prolongs dominance durations of the congruent visual stimulus and is also able to rescue it from suppression, making it visible (Lunghi et al., 2010). This cross-modal interaction is highly specific and relies on the objective congruency in spatial frequency (Lunghi et al., 2010), orientation (Lunghi & Alais, 2013), and spatial proximity (Lunghi & Morrone, 2013) between the visual image and the tactile stimulus. A study investigating the effect of touch on the strength of binocular rivalry suppression using visual and tactile gratings showed that congruent haptic stimulation interacted with binocular rivalry mainly by preventing the congruent visual stimulus from becoming deeply suppressed (Lunghi & Alais, 2015). While this suggests the cross-modal interaction involves strengthening the congruent visual stimulus, a complementary possibility yet to be investigated is that the tactile stimulus may also suppress the incongruent visual stimulus. Even though these two mechanisms are not mutually exclusive, binocular rivalry paradigms (unlike CFS) cannot disentangle their relative contribution to the observed effect. Understanding this is particularly important for assessing whether the integration between visual and haptic signals occurs at the conscious level (through the suppression of the incongruent dominant stimulus) or unconscious level (through the enhancement of the suppressed congruent visual signal).

Here, we investigate whether haptic stimulation can interact with a visual grating stimulus rendered invisible by CFS. Using a tactile grating stimulus, we will test whether the interaction with the suppressed visual grating is tuned for matching spatial frequency and orientation. On the basis of the results obtained in binocular rivalry paradigms, we anticipate that haptic gratings, when congruent with orientation and spatial frequency of the visual grating, will decrease the suppression duration of the visual grating.

## Materials and Methods

### Subjects

A total of 14 subjects (3 females, average age ± *SD*: 25.9 ± 3.5 years), including two authors of this study (C. L., and L. L.), participated in the experiment. All subjects had normal or corrected-to-normal vision, no strong eye preference (measured as eye-predominance in binocular rivalry), normal stereoacuity (Frisby Stereotest, Sasieni, 1978) and were right-handed. Except the two authors, all subjects were naïve to the purposes of the experiment.

### Ethic Statement

The experimental protocol was approved by the Tuscany regional ethics committee of the Azienda Ospedaliero-Universitaria Meyer and was performed in accordance with the *Declaration of Helsinki*. All participants gave written informed consent.

### Apparatus and Stimuli

**Figure 1.**
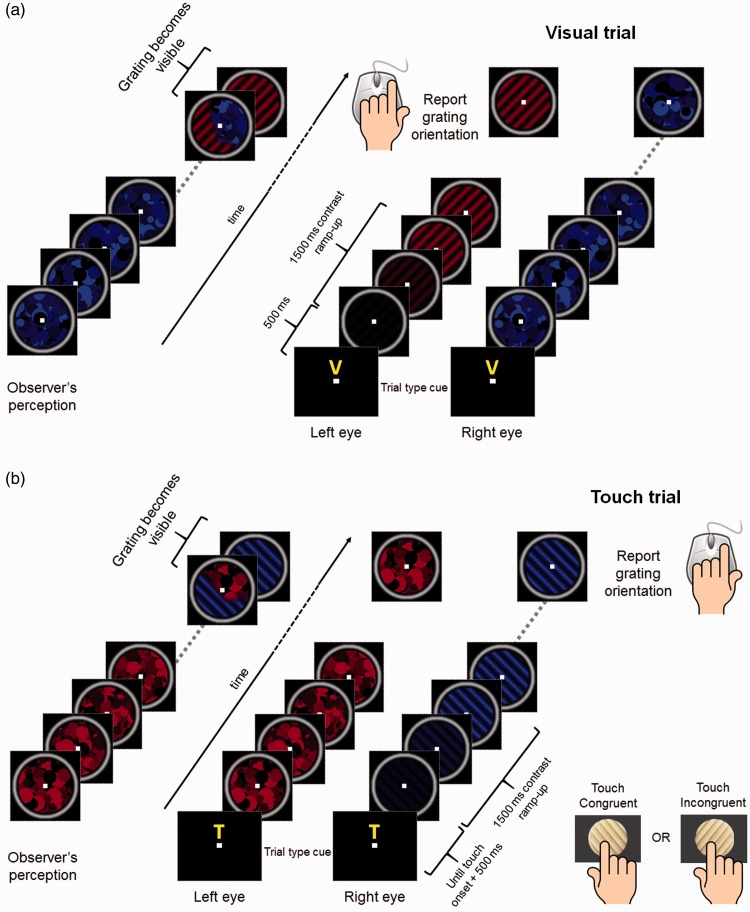
Experimental paradigm. (a) Diagram of a visual trial: 500 ms after the onset of the cue (letter “V”), the visual stimuli appeared. The contrast of the target grating was ramped from 0 to 10%, 20%, or 40% to avoid any transient that could break CFS. When the grating emerged from interocular suppression, observers reported its orientation (tilted left or tilted right) by pressing the appropriate mouse button. (b) Diagram of a visuo-haptic trial: same as (a), except that after the presentation of the cue observers were instructed to reach the haptic grating with their right index finger and explore it until the visual grating became visible. The visual stimuli appeared 500 ms after the onset of touch. On each trial, the orientation of the haptic grating was varied and could be either congruent or incongruent with the orientation of the visual target grating.

The experiment took place in a quiet room in total darkness to remove any spatial frame of reference. The visual stimuli were developed in Matlab (version 7.11.0, The MathWorks Inc., Natick, MA) using Psychtoolbox-3 (Brainard, 1997; Pelli, 1997) running on a PC. The stimuli consisted of red or blue dynamic maskers (“Mondrians,” Hesselmann & Malach, 2011) and red or blue target gratings. The stimuli were presented dichoptically through anaglyph red and blue goggles, superimposed with the grating in the center of the Mondrian, and were presented on a uniform black background (luminance: 0.17 cd/m^2^) in central vision, surrounded by a white smoothed circle (see Figure 1). The target stimulus was one of two orthogonal sinusoidal gratings (orientation: ±45°, diameter: 3°). In separate blocks, gratings having different contrasts (10%, 20%, or 40% of the maximum contrast) and spatial frequencies (2 or 3 cycles/degree) were used as target stimuli. Peak luminance of the red stimulus was matched to the peak luminance of the blue one (1.7 cd/m^2^). A set of 50 independent Mondrian-like masks were pregenerated and served as suppressors. These were presented to one eye at a rate of 10 Hz, meaning that the suppressor was refreshed with an independently drawn suppressor from the set of Mondrians every 100 ms. Mondrian masks were generated by randomly positioning 64 minimum, maximum, and medium luminance red or blue circles of different size (ranging from 0.12° to 0.54°). For an example of the visual stimuli used in the experiment, see Figure 1. The visual stimuli in each eye contained a small (0.2°) white fixation square in their center and were presented on a 24-inch LCD display (Acer LCD GD245HQ) positioned horizontally and mounted 35 cm above a mirror, so that the visual stimulus could be projected exactly over the location of the haptic stimulus. Observers viewed the stimuli reflected in the mirror, at a distance of 35 cm from the observers’ eyes.

Placed 35 cm under the mirror, the haptic stimulus consisted of a 3D-printed sinusoidal grating (diameter 3 cm, 2 cycles/cm of spatial frequency). The grating was mounted on a servomotor which could rotate the grating arbitrarily to any given orientation. We recorded the temporal dynamics of the movements of the observers’ fingers on the haptic grating using an LED and a photoresistor which were placed laterally to the 3D grating. When the observer’s finger reached the grating, the light beam from the LED to the photoresistor was interrupted, signaling the onset of touch. The servomotor, LED, and photoresistor were controlled by an Arduino UNO R3 microcontroller (D’Ausilio, 2012) interfaced with Matlab using the Arduino IO Package (http://www.mathworks.com/matlabcentral/fileexchange/32374-legacy-matlab-and-simulink-support-for-arduino).

### Experimental Procedure

Five different conditions were tested. Three experimental conditions tested visual target contrasts of 10%, 20%, and 40%. These conditions contained three types of trials: congruent touch, incongruent touch, and no touch. In all experimental conditions, the tactile and visual gratings matched in spatial frequency, but congruent (parallel) and incongruent (orthogonal) orientations were both compared against no-touch trials. For the current experiment, we define visual and haptic stimuli as being “congruent” when their objective features are compatible (e.g., the visual and haptic stimuli have the same size, orientation, and spatial frequency), so that the visuo-haptic information can be interpreted as arising from the same object. There were also two control conditions tested at 20% visual target contrast, comparing visuo-haptic stimuli mismatched in orientation (same spatial frequency as vision, 2 cycles/cm, but the haptic gratings were rotated 15° away from parallel and orthogonal to the visual orientation) and visuo-haptic stimuli mismatched in spatial frequency (haptic gratings were perfectly parallel or orthogonal to the visual grating but incongruent trials used haptic gratings with 3 cycles/cm instead of 2 cycles/cm).

All conditions were tested in separate blocks, and the experimental procedure was the same in all conditions. Each observer participated in two experimental sessions for each of the five conditions. Each session consisted of 54 trials, so that each participant performed a total of 540 trials, divided into 10 experimental sessions. At the beginning of each trial, a cue letter (“V” or “T”) presented for 500 ms indicated to the observer, whether it was a visual (“V”) or touch (“T”) trial. In visual trials (Figure 1(a)), the dichoptic CFS stimuli (a grating target to one eye and a dynamic Mondrian masker to the other) appeared 500 ms after the onset of the cue. To ensure invisibility of the target grating, its contrast was ramped from 0 to the condition’s maximum contrast (10%, 20%, or 40%) over a period of 1.5 sec following an on-ramp with a half-Gaussian profile.

The observer’s task was to report the orientation of the visual grating (left or right, pressing the corresponding mouse button) as soon as it broke through CFS suppression and became visible. The observer’s button-press determined the end of the trial. The touch trials (Figure 1(b)) were similar to the visual trials except that after the cue presentation, observers were instructed to reach for the haptic grating with their right index finger and explore it performing lateral scanning movements until the visual target grating became visible. On each trial, the haptic grating could be either congruent (parallel) with the visual target stimulus, or incongruent (orthogonal) with it. In touch trials, the visual stimuli appeared 500 ms after the onset of touch, as signaled by the photoresistor and LED combination. Both visual and touch trials were intermingled during each experimental session so that for each block similar numbers of visual, touch-congruent, and touch-incongruent trials were tested. The orientation of the target grating and the color (and therefore eye) associated with each visual stimulus (red or blue) was randomized on each trial.

### Exclusion of Subjects

A total of 2 of 14 subjects were excluded from the analyses because of poor accuracy (<75% correct responses) in the grating orientation discrimination task, indicating that these subjects were non-reliable.

### Analyses

Suppression times were computed for each stimulation type (visual, visuo-haptic congruent, visuo-haptic incongruent) and each experimental condition as the mean time elapsed between the visual stimulus presentation and the reported visibility of the grating. Trials in which observers reported the wrong visual grating orientation were discarded from the analyses. Outlier durations were discarded from the analyses, with outliers defined as suppression durations greater than ±3 *SD* from the average duration for each subject (15% of trials, on average). Accuracy in the visual task was computed as the percentage of correct responses. Suppression durations and accuracy for the different types of stimulation and each condition were compared across subjects using repeated-measures ANOVAs and two-tailed paired-samples *t* tests. The Bonferroni-Holm correction for multiple comparisons was applied to the *t* tests comparing different conditions.

## Results

### Main Experiment

**Figure 2.**
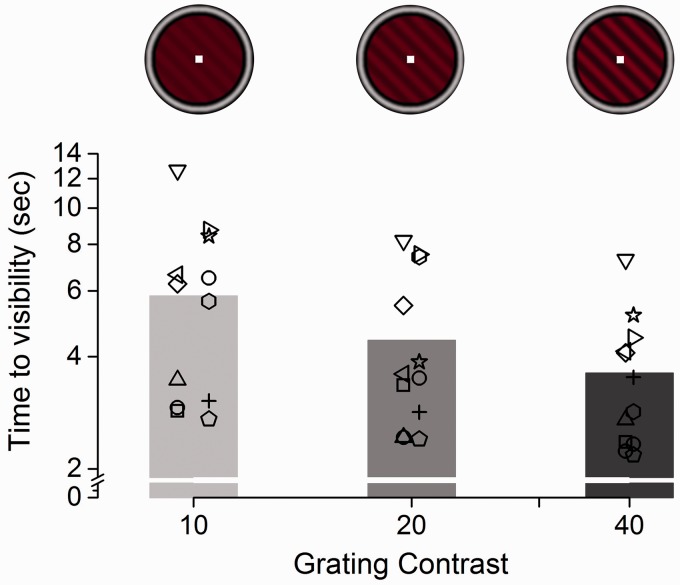
Effect of target stimulus contrast on suppression duration. Average suppression duration measured across trials (pooling visual-only and both visuo-haptic congruency conditions) for the three different contrast levels of the visual target grating. The symbols represent individual subjects’ suppression times across conditions. Suppression duration decreases linearly with increasing contrast of the target stimulus.

We investigated the effect of haptic stimulation on interocular suppression times combining CFS, in which dynamic Mondrian maskers presented to one eye suppress a visual grating (the target stimulus) in the other, with active exploration of a haptic grating congruent or incongruent in orientation with the visual grating (see Figure 1). Accuracy in the orientation discrimination task was very high across the group of 12 analyzed subjects, with a mean proportion correct (±1 *SEM*) of 0.98 ± 0.004. Erroneous trials (∼2% of all trials) were discarded from the analyses. In different blocks, visual stimuli having three different contrast levels (10%, 20%, or 40%) were tested. A 3 (contrast) × 3 (touch stimulation: no touch [visual-only], visuo-haptic congruent and incongruent) repeated-measures ANOVA revealed a significant main effect of the contrast factor, *F*(2, 22) = 10.25, *p* = .001, η^2 ^= 0.48, a significant main effect of the touch factor, *F*(2, 22) = 6.71, Greenhouse-Geisser corrected *p* = .02, η^2 ^= 0.38. There was no significant contrast × touch interaction, *F*(4, 44) = 1.77, *p* = .153, η^2 ^= 0.14. Polynomial tests revealed a strong linear trend for the contrast factor, *F*(1, 11) = 24.42, *p* < .0001, η^2 ^= 0.69, reflecting the linear decrease in suppression time with increasing contrast (Figure 2).

**Figure 3.**
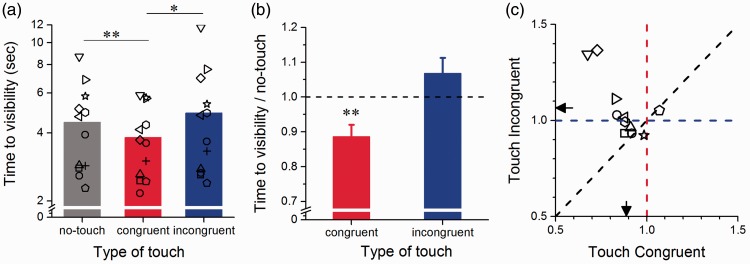
Effect of haptic stimulation on suppression durations. (a) Average suppression durations observed for the different types of stimulation averaged across visual target contrast levels: visual (no-touch, grey bar), congruent (red bar) and incongruent (blue bar) visuo-haptic stimulation. Error bars represent 1 ± SEM. Average (b) and individual (c) suppression durations (averaged across contrast levels) measured during visuo-haptic stimulation and normalized to durations measured during visual-only stimulation. (b) Normalized suppression durations are significantly shorter for congruent visuo-haptic stimulation (red bar), while durations for incongruent visuo-haptic stimulation (blue bar) do not differ from one. (c) The vertical and the horizontal dashed lines at the value 1, represent the case in which suppression durations do not differ from visual-only stimulation. The arrows indicate the average durations for congruent (red) and incongruent (blue) visuo-haptic stimulation. The symbols represent individual subjects’ suppression times across conditions. Asterisks indicate statistical significance in a paired-samples *t* test: **indicates *p* ≤ .01, *indicates *p* ≤ .01.

Suppression times also varied as a function of touch stimulation (Figure 3). Suppression durations were significantly shorter for congruent visuo-haptic stimulation (mean duration ±1 SEM = 3.81 ± 0.39 s) compared with both no-touch (mean duration ± 1 SEM = 4.45 ± 0.57 s: paired-samples *t* test, *t*(11) = 3.33, Bonferroni-Holm corrected α = 0.0167, *p* = .007) and to incongruent visuo-haptic stimulation (mean duration ± 1 SEM = 4.89 ± 0. sec: paired-samples *t* test, *t*(11) = 2.67, Bonferroni-Holm corrected α = 0.025, *p* = .022). There was no significant difference between no-touch and incongruent visuo-haptic stimulation (*p* > .05). Overall, suppression durations were 11% shorter for congruent visuo-haptic stimulation, compared with visual-only stimulation (mean suppression duration normalized to no-touch stimulation ± 1SEM = 0.89 ± 0.03, one-sample *t* test, H_0_: X = 1, *t*(11) = 3.40, Bonferroni-Holm corrected α = 0.025, *p* = .006). To represent the reduction of suppression durations induced by congruent visuo-haptic stimulation, Figure 3(b) (average) and 3(c) (individual subjects’ data) show suppression durations measured during congruent and incongruent visuo-haptic stimulation normalized for each subject to suppression durations obtained during visual-only stimulation.

**Figure 4.**
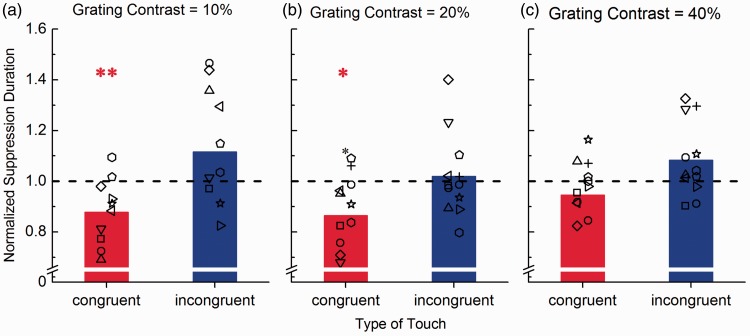
Normalized suppression durations for different target stimulus contrasts. Average suppression durations measured during congruent (red bars) and incongruent (blue bars) visuo-haptic stimulation normalized to durations measured during visual only stimulation. Different panels represent normalized durations measured separately for different visual target stimulus contrast levels: 10% (a), 20% (b), and 40% (c). The symbols represent individual subjects’ suppression times across conditions. Asterisks indicate statistical significance in a one-sample *t* test (H_0_: X = 1), *indicates *p* ≤ .05, **indicates *p* ≤ .01. There is a linear trend for a reduced effect of haptic stimulation on suppression durations with increasing visual target stimulus contrast.

The reduction in suppression duration for congruent visuo-haptic stimulation depended on the contrast of the visual target grating (Figure 4). For target stimulus contrasts of 10% and 20%, congruent touch significantly reduced suppression durations (10% contrast: −12%, one-sample *t* test, H_0_: X = 1, *t*(11) = 3.56, Bonferroni-Holm corrected α = 0.025, *p* = .005; 20% contrast: −13.5%, one-sample *t* test, H_0_: X = 1, *t*(11) = 3.05, Bonferroni-Holm corrected α = 0.025, *p* = .011). However, the effect was not significant for target stimulus contrast of 40% (−6%, one-sample *t* test, H_0_: X = 1, *t*(11) = 1.98, Bonferroni-Holm corrected α = 0.025, *p* = .073).

### Control Experiments

**Figure 5.**
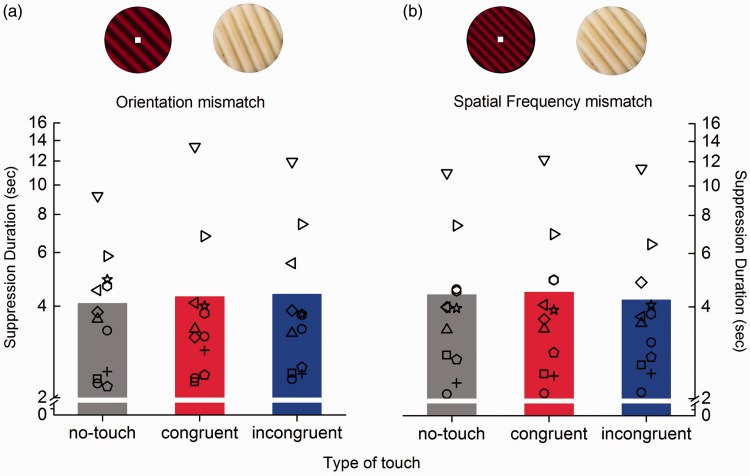
Control experiments: visuo-haptic orientation and spatial frequency mismatch. Average suppression durations measured in two control experiments in which a mismatch in either visuo-haptic orientation (a) or spatial frequency (b) was introduced. In both experiments, suppression durations measured either during congruent (red bars) or incongruent (blue bars) visuo-haptic stimulation did not differ from durations measured during visual only stimulation (no touch, grey bars). The symbols represent individual subjects’ suppression times across conditions.

Control experiments investigated whether the facilitatory effect of congruent haptic stimulation on visual suppression duration was selective for orientation and spatial frequency. We tested the effect of introducing a visuo-haptic mismatch between the gratings in either orientation (Figure 5(a): spatial frequency was matched, but +15° was added to the haptic gratings for both parallel and orthogonal conditions) or spatial frequency (Figure 5(b): parallel and orthogonal haptic gratings were as for the experimental condition, but haptic frequency was increased to 3 cycles/cm, vs. 2 cycles/cm for vision). The visual grating contrast was fixed at 20%. Whether the haptic grating mismatched with vision in orientation (Figure 5(a)) or in spatial frequency (Figure 5(b)), suppression durations were very similar. In both experiments, neither of the touch conditions (congruent vs. incongruent) differed from each other or from the no-touch condition (all *p*s > .41). This indicates that the effect of congruent touch on reducing CFS suppression durations is selective for both orientation and spatial frequency.

## Discussion

We have demonstrated that haptic stimulation is able to boost a congruent, suppressed visual stimulus into visual awareness. Exploring a haptic stimulus matching in orientation and spatial frequency with a visual stimulus rendered invisible by CFS shortened visual suppression duration. A number of recent studies have shown that signals in various non-visual sensory modalities can modulate the visibility of a visual stimulus during CFS. For example, visual suppression durations have been shown to be slightly shorter (in the range of 12–200 ms) when paired with congruent acoustic (Aller, Giani, Conrad, Watanabe, & Noppeney, 2015; Alsius & Munhall, 2013), proprioceptive (Salomon et al., 2013), olfactory (Zhou et al., 2010), vestibular (Salomon et al., 2015), and bodily (Salomon et al., 2016) signals*.* A limitation of these studies, however, is that they only compared suppression durations for congruent cross-modal stimuli with suppression durations for incongruent cross-modal stimuli. While these congruency effects suffice to reveal cross-modal interactions, the absence of a visual-only baseline makes it impossible to determine whether the cross-modal effects arise from a facilitation due to congruent cross-modal stimulation, or rather from a masking effect of the incongruent cross-modal stimulation. Here, we have demonstrated for the first time that cross-modal stimulation can actually speed the access of the suppressed visual stimulus to consciousness, relative to a visual-only condition. Our results show a robust decrease of CFS suppression durations during congruent visuo-haptic stimulation both compared with visual-only stimulation (−640 ms) and to incongruent visuo-haptic stimulation (−1008 ms), indicating unambiguously that congruent touch accelerates the rise of the suppressed image to visual awareness.

Our results are in line with accumulating recent evidence in binocular rivalry showing that touch can interact with a congruent visual stimulus when it is suppressed and shorten its suppression duration (Lunghi & Alais, 2013, 2015; Lunghi et al., 2010; Lunghi & Morrone, 2013; Lunghi et al., 2014). Here, using CFS, we provide an even stronger demonstration that congruent haptic and visual signals can interact outside of visual awareness. This is due to that fact that the initial period of perceptual suppression in CFS is complete: the dynamic mask totally suppresses the visual target stimulus, so that the observer cannot know the orientation or spatial frequency of the suppressed grating. During binocular rivalry, by contrast, visual perception oscillates between the two monocular images (e.g., orthogonal gratings), so that even though one stimulus may be clearly dominant at a given moment (and thus the other suppressed), the observer has some cognitive knowledge about the features of the suppressed visual stimulus and has likely seen it in the previous rivalry phase. This knowledge of the suppressed stimulus’ properties could potentially contribute to the congruency effect of cross-modal stimulation in binocular rivalry. By replicating the effects using CFS, we rule out the possibility that they are entirely mediated by response bias or shifts in cross-modal attention. This is also corroborated by the strict selectivity of the effect that we found for matched visuo-haptic orientations and spatial frequencies. In fact, it has been previously reported (Lunghi & Alais, 2013) that when engaged in a visual task, observers cannot consciously recognize a visuo-haptic orientation mismatch of 15° (that in our experiment disrupts the cross-modal interaction). This further supports the evidence that the facilitatory effect of touch that we found is not attributable to a high-level semantic priming effect or categorical decision of the observer.

Another strength of the CFS paradigm for demonstrating cross-modal interactions is that the target grating rivals with an unrelated dynamic Mondrian mask. In the binocular rivalry studies mentioned earlier, visual competition is often created by dichoptically presenting orthogonal gratings, so that the suppression of the visual grating congruent with the haptic orientation corresponds to the dominance phase of the visual grating incongruent with the haptic orientation. This makes it difficult to completely exclude the possibility that the shorter suppression durations observed during congruent visuo-haptic stimulation are in fact due to a concomitant inhibitory effect of touch on the dominant incongruent visual grating and thereby reducing its dominance duration. In CFS, however, the visual grating congruent in orientation with the haptic one is suppressed by a dynamic Mondrian mask that is completely unrelated to the visual target stimulus. Therefore, we can conclude with confidence that the facilitatory effect of congruent cross-modal stimulation is due to touch strongly boosting the strength of the suppressed visual signal. Indeed, based on previous measures of the strength of CFS suppression, the boost in strength of the suppressed visual image by the congruent tactile grating must be considerable because contrast sensitivity during CFS is reduced enormously, about a 20-fold decrease relative to monocular viewing (Tsuchiya et al., 2006). The vigorous effect of congruent haptic stimulation on suppressed visual stimuli indicates that signals arising from different sensory modalities can enhance visual awareness. This suggests that unisensory awareness is not encapsulated for each modality, but rather that human awareness should be considered as a multisensory phenomenon, in which conscious perception in one sensory modality (e.g., touch) can enhance awareness in a different one (e.g., vision). This cross-modal boost of visual awareness relies on congruency between the stimuli in the different modalities (Deroy et al., 2016), whereas it is not observed for newly learnt cross-modal associations (Faivre, Mudrik, Schwartz, & Koch, 2014).

Another interesting aspect of these data is the evidence for stimulus selectivity in the cross-modal interaction. Our control experiments showed that the effect of touch on CFS disappeared when we introduced a small mismatch in visuo-haptic orientation (+15°) and a small difference (less than one octave) in visuo-haptic spatial frequency. This indicates that the interaction between touch and vision is specific and tightly tuned for these two basic visual features, a result that is in line with previous visuo-haptic studies of binocular rivalry (Lunghi & Alais, 2013; Lunghi et al., 2010; Lunghi & Morrone, 2013) and with recent work on visuo-haptic contrast discrimination (van der Groen, van der Burg, Lunghi, & Alais, 2013). It is well known that such a tight selectivity both for orientation and spatial frequency is a unique property of neurons in the primary visual cortex (Hubel, Wiesel, & Stryker, 1977; Maffei & Fiorentini, 1973). In the present experiment, the position of the hand was vertically aligned with the haptic stimulus, in order to allow a body-referred reference frame for the tactile orientation processing, and a clear consistency with the visual reference frame. It is however possible that shifting the haptic reference frame by changing the position of the hand to an unnatural one might lead to different results.

Together with evidence showing that CFS occurs early in the visual system, so that neural activity (BOLD) of visual stimuli suppressed by CFS is reduced already in the primary visual cortex (Yuval-Greenberg & Heeger, 2013), our results strongly suggest that haptic signals can interact with visual signals already at the earliest stages of visual processing in a stimulus-selective manner.
